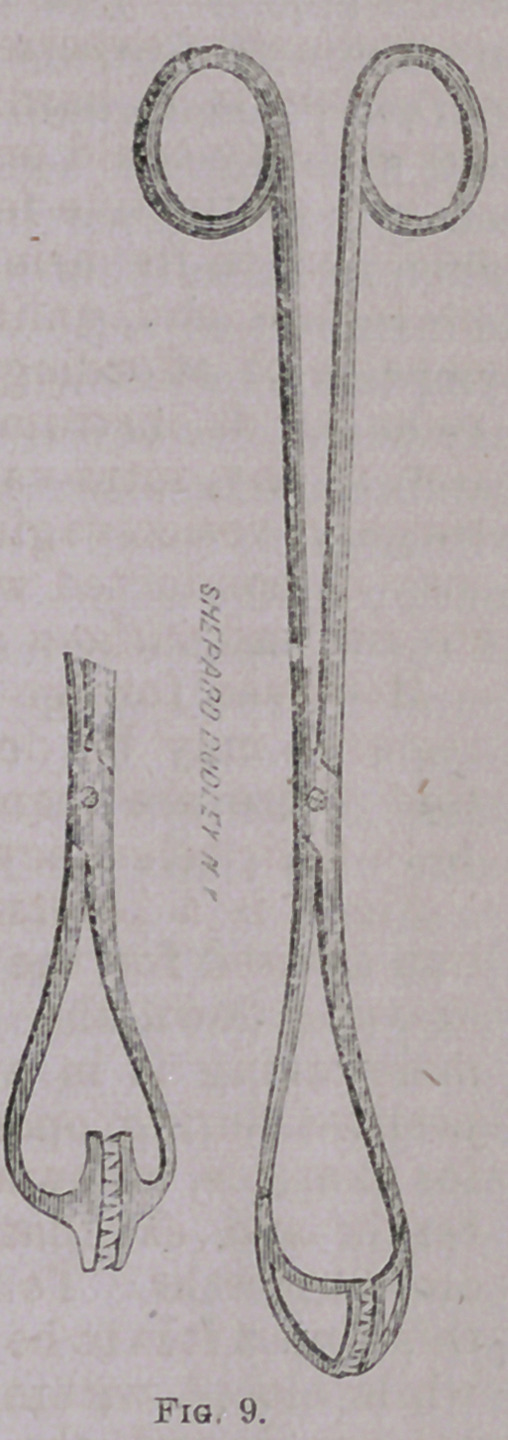# Clinical Notes on the Electric Cautery in Uterine Surgery

**Published:** 1872-12

**Authors:** J. Byrne

**Affiliations:** Surgeon-in-Chief to St. Mary’s Hospital for Diseases of Women; Clinical Professor of Uterine Surgery to Long Island Medical College, etc.


### Miscellaneous.

### Clinical Notes of the Electric Cautery in Uterine Surgery.

By J. Byrne, M. D., Surgeon-in-Chief to St. Mary's Hospital for Diseases of Women; Clinical Professor of Uterine Surgery to Long Island Medical College, etc.

A few months ago, at a meeting of the New York Obstetrical Society, Dr. Chamberlain reported a case of epithelioma of the cervix uteri in which, though the affected part had been very satisfactorily removed by galvano-cautery, the disease had nevertheless reappeared within five or six weeks after the operation. Other instances also were referred to, where an equally unsatisfactory result had followed this method of operating; and the prevailing opinion of members present appeared to be that the removal of such outgrowths from these parts, even by the cautery, offered but little encouragement as a curative measure, and that reported successes were at best exceptional, or of rare occurrence, if not doubtful as to diagnostic accuracy.

Indeed, whether on account of disheartening experiences then related, or the well-known difficulties attending the management of galvanic batteries, there seemed to be, if not a disposition to doubt the utility of resorting to any operation in such cases, at least a strong desire to have the subject more fully presented. With a view to supply this want in some measure, and especially as I had referred more encouragingly to my own observations in the use of the galvanic cautery, I was requested to furnish a paper, which was read at a meeting of the Society held in June last.

Experiments undertaken over two years ago, and noticed on the occasion referred to, have been steadily continued during this interval, and cases of great interest, in which I have operated by this means, have since presented themselves.

Thus, while I have succeeded in devising a compact and effective galvannic battery suited to every surgical emergency, and yet comparatively simple in its management, together with improved electrodes and platina instruments, ample opportunities have been afforded from time to time for practically testing the value of each novelty as suggested. This is my apology for delaying the publication of my paper until this time.

No surgeon who, having witnessed for the first time a successful intra-vaginal operation by the galvanic cautery,—for example, the removal of a cualiflower cancer, or a fibrous polypus from the cervix uteri—can have failed to appreciate the many advantages offered by this safe and rapid, yet bloodless proceeding, over all other means heretofore at our command. He might also feel astonished that so admirable a method of conducting these and similar operations had not been more generally adopted by gynæcologists especially, or the subject even assigned a few pages in works on that speciality; for a late edition of one of the most practical, if not the very best treatise on diseases of women, is in this particular noticeably defective.*

A very little reflection, however, will soon convince him that, after all, neither authors or any one class of practitioners in particular are much to blame; for even the laws by which galvanic electricity is governed, not to speak of its adaptation to the most delicate and difficult operations, are nowadays but seldom made the subject of scientific inquiry either by candidates for medical honors or practitioners generally. And yet, strange as it may appear, the history of galvano-cautery, though consisting for the most part of clinical fragments merely, or an occasional report of some chirurgical achievement, covers a period of over a quarter of a century. It is true, but little was heard during the first few years of the new service which the thermal power of current electricity was being made to render; for prior to 1850 almost the only surgical uses which it seems to have served, with the exception of Crussel's operation for a fungus hæmatodes, were the removal of nævi, and the destruction of dental nerves It may be safely asserted, however, that we are indebted for most of what is even yet known of galvano-cautery to the ingenious devises of Marshall and Ellis, in England, from 1850 to 1852, and the subsequent publication, in 1854, of Mideldorpff's more brilliant exploits in Germany. Since the latter period, many interesting reports in cases by Semeleder, Newman, Zsigmondy, Braun, Von Grenewald, Rudolph, Voltolini, and others, have appeared, but there is nothing in the valuable yet only corroborative experiences of these observers, to warrant a doubt that the claim of priority in originating all that is of practical value in electro-cautery, belongs by right to those first named.* To Ellis, especially, is due the credit of first suggesting the spiral cauterizer; Marshall and Middeldorpff, contemporaneously, though independently, devised the loop; while all clearly and distinctly indicated the various lesions likely to be benefited or cured by the employment of their several contrivances.

It is not a little surprising, therefore, to notice how few surgeons there are, comparatively, even among gynæcologists, who have adopted the practice, or given the subject any attention whatever, though more than twenty years have now elapsed since its claims were so attractively demonstrated. That this omission arises, in a great measure, from the want of any reliable guide to a practical

*The two pages devoted to galvano-cautery in the work of Professor Thomas, to which I refer, must have at least one good effect,—that of inciting the reader's desire to know something more of the subject.

* In this country, also, many important galvano-cautery operations has been performed within the last few years by Drs Noeggerath, Thomas Guleke, Sims, Jacobi, and others, but few of which, however, have been published, so far as I know.

study of the subject, there can be little doubt; because, as has already been intimated, any one who desires accurate information, or such definite rules and directions as will enable him to operate successfully by means of the electric cautery, will seek such aid in vain among the gynæcological records, or other medical literature of our language at least. The brief allusions met with in standard works on medical electricity and electrolytic surgery, will avail but little in a practical sense, beyond what relates to the elementary principles of electro-physics. As for practical hints, and that particular kind of knowledge so needed for conducting important cautery operations, there are but two ways in which such can be obtained: either by being fortunate enough to have repeatedly witnessed and closely observed such operations, or through laborious experimental research and no trifling pecuniary outlay. By this latter path I have been obliged to travel; and though fortified by a tolerably exact knowledge of electro-physics, and constantly aided by material for clinical study, yet many disappointments, and difficulties of a perplexing nature have had to be at first contended against.

This statement is made, not with a view to herald my own industry or perseverance, but merely as suggestive of additional reasons why galvano-cautery, which is destined at no distant period to play a most important part in gynæcological practice, is so little understood, and so seldom resorted to. It is also reasonable to infer from what has been said, that many of the unsuccessful attempts to operate by galvano-cautery of which we hear, as for example when the battery is said to have "given out" at a critical moment, have been due less to imperfections in the apparatus, than a want of experience and inadequate knowledge of electro-physics on the part of the operator.

It will be found impossible to construct any galvano-electric apparatus which may not occasionally become defective, either by accidental displacement of some of its parts, or imperfections resulting from use. The well-ascertained laws, also, in accordance with which the electric fluid is generated and set in motion, demand the strictest observance, and will tolerate no innovations incompatible therewith, either as regards the relation of negative and positive elements to each other, and their metalic connections, or the quantity and kind of fluid or fluids by the aid of which electro-motive force is to be obtained.

Consequently no surgeon can hope to succeed in the practice of electro-cautery unless, when difficulties arise, as in case of failing to obtain sufficient heat, he is not only competent to fully appreciate and understand the nature, causes, and extent of such interruptions, but also possessed of a certain amount of mechanical aptitude so as to enable him to remedy the defect Indeed I have no hesitation in stating that these conditions are essential to success, and cannot be safely dispensed with; because, though certain rules may be laid down concerning the general management of batteries, and even specific directions given as to the proper manner of conducting cautery operations, nothing short of a tolerably exact scientific knowledge of the whole subject will suffice to overcome unavoidable obstacles.

Hence, it is not unreasonable to infer, that had these facts been earlier recognized, many of the troubles and disappointments reported in the practice of eminent surgeons might have been avoided, nor would a quarter of a century have elapsed ere galvano-cautery, instead of being understood and practiced by comparatively few, had become the usual, and not the exceptional means by which certain diseased conditions might be cured or relieved.

Before proceeding to describe such a battery and instruments as I have found best suited to the requirements of surgical practice, some reference to the several kinds of galvanic apparatus used and recommended by others seemed called for. Nearly three years ago I assisted Dr. Noeggerath in removing an epithelioma from the cervix uteri of a lady whose case will be described hereafter, and the battery used on that occasion was a zinc-carbon one, such as that first invented by Bunsen in 1843. I subsequently operated with this instrument, and was much pleased with its action in both cases, though in the latter my patient, who had a large fibro-cellural polypus attached by a thick pedicle, lost much blood, owing to the vascularity of the parts, but more particularly because the wire used was, as I believe, too fine, and perhaps also in some measure on account of traction kept up on the tumor. I remarked then to gentlemen present that vascular parts could not be safely cut through except by a much thicker wire, which I was informed the battery, though a very large one, would not sufficiently heat.

My next few operations were conducted by means of a powerful Grove battery, the only distinctive difference being that platina instead of carbon is used as a negative element, and in every respect similar to that used by Professor Middeldorpff.

This apparatus, though beautifully constructed and costly, was soon abandoned, however, mainly because of the great trouble and care needed in working it; for like the one first used, strong nitric acid was required for the inner or porous cell, and on account of which perplexing accidents are often unavoidable. Nevertheless, being favorably impressed by what I had already observed, and influenced by the opinions of authorities against other than constant batteries, I determined to provide myself with another 8-cell Bunsen, similar in principle to that of Dr. Noeggerath, already referred to. After a few trials, however, I found it quite insufficient to heat wire of such length and thickness as would insure against hemorrhage in any but trifling operations. This defect, coupled with the danger in handling large quantities of strong nitric acid, and the suffocating nitrous fumes resulting from chemical action, not to speak of the trouble and time spent in filling, emptying, and cleaning the cells, induced me to abandon every kind of so called constant, or two-fluid battery.

The claims of Stohrer's four-cell one-fluid carbon-zinc battery were next fully considered, but on account of its huge dimensions, being less portable than any of those already tried, I hesitated, and concluded to procure the French contrivance, known as the "Grenet battery." This little apparatus is composed of eight zinc and six carbon plates, four of the former being united and connected with three of the latter, similarly joined, the other sets of three and four zincs and carbons, each unitedly forming the negative and positive poles.

In this manner the whole is made to act as *two* powerful cells. I have operated frequently with this instrument, and can fully endorse the views expressed regarding its power and certainty of action by Dr. Garret, of Boston, the only author, so far as I know, whose opinious as to its worth seem to have been derived from a practical knowledge of its capacity. After an extensive practical acquaintance with this battery it is a little amusing to recall the description given of it by Meyer, in his work on "ELECTRICITY IN ITS RELATIONS TO PRACTICAL MEDICINE," as follows:—"After the battery is dipped into the fluid *as high as the upper edge of the carbon plates*, a Y-shaped tube is fastened to the rubber tube, and to this a pair of bellows; soon the fluid is thrown into commotion, and after *four or five seconds* the platinum wire which is secured to the conducting wires going from the zinc and carbon poles, glows." None of which is correct, because neither this nor any other such battery should ever be dipped "as high as the upper edge of the carbon-plates," no bellows is needed, and the platina requires no longer time to become incandescent than when attached to any other battery that I have ever seen. I have never found it necessary to use the bellows attachment, the occasional raising and re-immersion of the battery being all that is needed to perpetuate its power. It has not, however, that "*intensity*" arrangement which many operations demand, and hence its sphere of action is too limited to be universally serviceable in practice. Moreover the lead-lined box which contains the fluid is too large to be conveniently portable, and there is no mechanism provided for raising the battery out of the acid solution when not actually in use, and keeping it suspended so as to drain the plates, arrest chemical action, and thereby control and preserve its heating capacity.

Being on the whole, tolerably well satisfied with this first specimen of single-fluid battery, my next desire was to obtain one of a similar nature, but, if possible, still more powerful and less limited in its sphere of action, yet as moderate in size as would be consistent with these additional requirements.

A battery combining in a very great degree all these qualities was therefore constructed at my request by Mr. Charles T. Chester, 104 Centre street, whose thorough practical acquaintance with electro-physics is so well known, and to whose politeness I am greatly indebted for many valuable hints and suggestions. This instrument is composed of eight pairs of carbon and zinc plates, each measuring about six by nine inches, and so arranged that the whole could be made to act either as two cells when *quantity* is desired, or four cells as when greater *intensity* is needed to overcome resistance. If short and heavy, or flattened platina is to be heated, certain binding screws marked two or turned down, while those marked four are to be raised; and when a long and comparatively thin wire, such as is used for looping purposes, is required, this order of adjustment is to be reversed. By this useful contrivance the apparatus can be made to meet every want, and in my hands it has never failed. As an evidence of its power, moreover, I may state that five inches of number sixteen wire can be made incandescent, and as the elements can easily be raised or lowered by means of a windlass attachment, its management is simple, and as a whole it is far superior, in my estimation, to the more clumsy and costly apparatus of Stohrer.

With all these attractive features, however, it also is too bulky and heavy to be conveniently portable, and consequently not so well adapted to the requirements of private as to hospital practice. The quantity of fluid required to bring it into action is three gallons, prepared by dissolving three pounds of bichromate of potassa in ten quarts of boiling water, to which, when cool, two quarts of sulphuric acid are to be added.

It will be observed from these remarks that, though double-celled batteries, whether composed of the Bunsen or Grove elements, are constant in their action, they possess no other attractive characteristic warranting a preference over the more simple and manageble single-fluid arrangements. Indeed, this supposed indispensable quality as to constancy may be conveniently dispensed with, for it is no more an essential requisite in a battery for surgical purposes, than would be perpetual motion in a time-piece.

Authorities on electro-surgery, as a rule, either caution us against the employment of these batteries simply because they are not continuous in their action, and liable to give out at a critical moment, or furnish such an incorrect description of their *modus operandi* as to deter many from using them. They seem to entirely forget, however, or at least fail to suggest that there can be no reasonable object whatever in immersing a battery before its action is called for, or allowing it to remain so unnecessarily long, and during intervals of inspection which ought to, and must occur during every important cautery operation. Another very common and mischievous fallacy is, to suppose that by disconnecting one or other of the conducting cords, or otherwise breaking the current, as, for example, by means of a slide in the cautery-handle, we thereby arrest the waste of thermal power. Breaking the current, however, does not wholly arrest chemical action; and as prolonged immersion, even with this precaution, seriously impairs electromotive power, no battery of this class should be put in contact with the fluid until heat is actually required, or allowed to remain so during operative interruptions, or one minute after it has served its purpose.

### DESCRIPTION OF BATTERY.

Fig. 1 is a correct representation of a battery devised by me over twelve months ago, and employed in some of my most important operations.

It consists of twelve carbons and twelve zincs, each 3 by 5 inches, combined and arranged so as to represent four sets or cells of three pairs each. In this order the elements are securely fastened by nuts and screws to a hard rubber platform 7½ by 8 inches in surface, and one-quarter inch thick; and the combinations and connections effected by means of narrow strips of copper annealed and nickel-plated. In the centre is a cog-wheel 3 inches in diameter, which, on being turned by means of an upright handle, causes the two water-agitators to revolve.* Near the front edge of the platform is fixed what might be properly denominated an *electro-motive*, or *electro-tension disc*, by the aid of which the whole character of the battery may be changed in a moment, so as to represent either two cells, as when quantity is needed, or four cells when great resistance is overcome, such as in heating a long thin wire.

FIG. 1.

The latter simple contrivance has rendered this battery, in my hands, equally reliable and powerful in every emergency, being capable of heating (white) from 6 to 8 inches of No. 16 wire (Stubb's gauge), or over 12 inches of No. 21, the last mentioned being the size which I always select for looping purposes. Passing through the centre is a square perpendicular rod notched on one side for the reception of a ratchet-spring fixed to the collar of the central wheel, and by which the battery may be easily lowered into the liquid, or raised and kept suspended at any point desired. This arrange-

* The object of this arrangement is to increase the power of the battery when, owing to continued use, as in tedious operations, the fluid may become exhausted.

It is very seldom needed, but as an example of its value under certain circumstances, I may state that half strength fluid—that is, one part of water and one of fresh ordinary battery fluid—can be made, by agitation in this manner, to produce nearly, if not quite, an equal heat with the strongest fluid without such agitation.

ment is much preferable to that of a screw, as in Stohrer's instrument, because the small size of my apparatus as compared with the former, enables the assistant in charge to regulate its power according to the demands of the operator, with less delay and equal facility.

The upright rod being screwed into a transverse support in the box, can be removed when the battery is not in use.

The box is divided into two parts by a central plate, suspended above, and running from before backwards; a stop-cock is provided for drawing off the fluid and washing out the battery after being used, and the whole being made of hard rubber moulded, there is no necessity for lead or other lining.

The conducting cords ought to consist of not less than 100 strands of fine copper, or what is still better, silver wire, each cord well covered with silk or cotton in the first place, and then, as a matter of great convenience when operating, bound together by another covering to commence 12 inches from the binding-screw extremities, and to continue up to within three inches of the opposite ends. The latter should each be provided with a socket and sliding ring for the reception of the cautery handles, as this is a much better and less bulky manner of making connection than by binding screws as ordinarily employed.*

### DIRECTIONS FOR PREPARING THE BATTERY.

The quantity of fluid required is six pints, prepared by dissolving twelve ounces of bichromate of potassa in five pints of boiling water, to which, *when cool*, one pint of sulphuric acid is to be slowly added. Owing to the chemical heat generated by the admixture of the acid, the liquid must again be allowed to cool before using; otherwise, the zinc plates would suffer much waste, and the efficiency of the whole apparatus then and for the future be seriously impaired. Every battery ought to be carefully examined *each time* before commencing operations with it, so as to make sure that every part is in order, and that no displacement or contact of zincs and carbons has taken place since last in use. Before pouring the fluid into the box, the elements should be lifted out carefully and rested on some smooth surface, and the quantity above stated (six pints) should be measured, unless, as I have suggested to the manufacturers, a mark be placed on the inside to indicate the required quantity. The next step will be to screw on the upright rod, and suspend the battery sufficiently high to be out of the bath. The conducting cords may next be adjusted, and in doing so, care should be taken that the binding screws are turned down tightly, so as to insure perfect connection, the same exactness being also necessary in regard to the handle attachments.

* This battery, as well as every form of electrode required, is manufactured by Shepard & Dudley, 150 William street; and for the perfect and satisfactory manner in which my instructions have been carried out, as to their construction, much credit is due to the good taste and mechanical judgment of Mr. William R. Leonard, with the above firm.

FIG. 2.

Figure 2 represents an improved loop instrument originally manufactured by Mr. Charles T. Chester at my suggestion, and is far superior to any other that I have seen used or described, for the following among other reasons: The loop is tightened by straight traction instead of being wound on a roller, and thus less likely to be impaired for future service; while the opera glass attachment enables the surgeon to keep up a more regular and steady action than would be possible by turning a wheel. Moreover, by using such an instrument as this he will be more likely to avoid the frequent and serious mistake of cutting through the tissues too rapidly, thereby forfeiting one of the main advantages justly claimed for galvano-cautery, which I need hardly say, is security against hemorrhage.*

Figure 3 is the spiral cauterizer which I have been in the habit of using successfully in cases of chronic inflammatory affections of the urethral mucous membrane, and as a more thorough, safe, and radical means of combating obstinate follicular disease of the cervical canal than any other caustic or stimulating application

FIG. 2.

heretofore employed or recommended. Within the last few months, however, I have devised and used what I consider a much better means of accomplishing the same purpose, by substituting for the spiral wire and porcelain, 5 inches of a heavier wire (say No. 16), flattened and doubled so as to nearly represent a long cylinder. In this manner the treatment here recommended may be very thoroughly carried out.

FIG. 4.

The cautery knife and handle are tolerably well shown in figure 4, and, as the uses to which the former is applicable will be referred to elsewhere, no description need be here given.

* The loop end of this instrument is someweat different from that exhibited in the drawing, and is provided with a wooden casing to protect the sound parts from injury by reflected heat in the metalic conductors.

So also in regard to the illustrations A B C D E F G; while some will be clinically noticed hereafter, the uses which each is designed to serve can hardly fail to be understood by a moment's reflection.

It may be proper to remark, however, that the dome-shaped cauterizer D is used for the purpose of searing over surfaces from which morbid growths may have been exterpated, or stopping the open mouths of bleeding vessels; and the little knife G is that delineated in figure 10, where one of the many useful purposes to which it may be applied is planly indicated.

In addition to the battery and electrodes herein described, it must not be forgotten that the operator will have to be provided with certain other contrivances designed especially to facilitate cautery operations, though as to their range of usefulness by no means limited to such purposes, as for example,

A SUITABLE SPECULUM.

Ordinary devices of this nature, though answering tolerably well for a mere occular examination of the cervix uteri, or routine topical treatment, will be of no service whatever for the purpose under consideration, because parts to which the actual cautery is to be applied must not only be brought well into view and within perfect control, but as far as possible isolated from surrounding structures. Besides, patients, whether anæsthetized or not, are often restless, and the slightest movement at a critical moment might seriously affect the whole subsequent proceedings, were not some provision made against all such contingencies.

Moreover, it must not be forgotten that inexperience on the part of an assistant, or the most trifling variation in the position of his hand, often rendered unavoidable by fatigue, may equally interfere with the operator's design.

If a Sims speculum be used, at least two experienced and reliable assistants will be needed, one to hold that instrument, and the other to take charge of the anterior vaginal wall, yet neither can render any other kind of aid while thus engaged. The strongest objection to its use, however, is the position in which a patient must necessarily be placed, for I contend that no uterine operation by galvano-cautery can be satisfactorily conducted unless the patient is made to assume the dorsal attitude.

Granting, then, that these views are in the main correct, and knowing from extensive clinical experience that we do possess a means by which most of the important desiderata here indicated may be obtained, any device combining properties so attractive, demands something more than a mere passing notice.

The instrument referred to, is the speculum introduced and described by me about fifteen months ago, and a modification of which is here shown* (Fig. 5).

FIG. 5.

This speculum, it will be observed, differs none in principle from that previously noticed, and, as to the several pieces of which it is composed, they may be considered the same, *with one exception*, namely—the frame on which the lower or perineal blade moves is much wider and a little longer, thereby affording more working space and greatly facilitating operative manipulations. The foreshortened view in the above sketch will serve to explain more clearly the points of difference between this "operating," and the ordinary speculum.

Some advantages, however, will be found by having the intra-vaginal parts of this instrument a little longer—say half an inch—and from one-quarter to three-eighths wider than the ordinary size. I have also occasionally resorted to a piece of bent spring wire, to be introduced after the speculum has been adjusted and the uterus fixed in position, for the purpose of still further separating the lateral walls. This, though by no means an indispensable requisite *in any case*, may nevertheless be made to render good service, under certain circumstances, and on this account I have given directions to have some such device supplied with each "*operating*" speculum.

Fig. 6 is intended to illustrate more clearly the principle on which this speculum is constructed, and the *modus operandi* by which the curved vaginal canal is not merely dilated, but straightened by pressing back the perineum BELOW, while the vesical wall is elevated ABOVE. The under blade, it will be noticed, is made to move in a circle of which the centre is indicated by its point, so that the relation of the latter to the cul-de-sac, when the instrument is first introduced, does not materially change, no matter to what extent the perineal blade may be pressed backward. The

* For a full description of this instrument *American Journal of Obstetrics and Diseases of Women and Children*, see for Aug. 1871.

Fig. 6.

various directions, too, in which the upper double rod may be made to move, is a most important feature in this instrument; for, however displaced a uterus may be, more especially if anteverted, and provided no firm adhesions exist, there is no difficulty in bringing it into view, and so fixing it for examination or treatment.

The difficulties said to have been met with by some in using this instrument may, I think, be very readily accounted for, and I would submit the following as the most probable and rational explanation: In the first place it has been found almost impossible, up to a very recent period, to get manufacturers to carry out my instructions as to its mechanism, and the consequence has been that quite a large number of imperfect instruments have found their way into the hands of practitioners. That this has been a source of serious annoyance and much disappointment there can be no doubt, for I have myself seen more than one worthless specimen; and wherever I have had the opportunity, have insisted on the purchaser's returning it. This drawback, I am told by the various makers, is now at an end, and there will be no difficulty for the future in obtaining the perfect instrument. Nevertheless, every purchaser should carefully examine to see that the principle as to circular motion, &c., is carried and that *the width of the upper blade is rather less than that of the lower.**

Again it not unfrequently happens that some physicians undertake to use it without reflecting on the purposes for which it has been devised, or the directions heretofore given for its application, and as a natural consequence often blunder in adjusting it. There are others, too, I am told, who seem to have been disappointed at

* Say not more than one inch and a quarter, outside measurement.

failing to find in this contrivance an automatic speculum, by the aid of which common sense and ordinary judgment in uterine examinations might safely be dispensed with. One of the latter class, if asked his opinion of it, will very likely reply that he could not possibly get along with it, as in his hands it caused much pain to the patient, and after all offers no advantages that he can see over any one of half-a-dozen others. Akin to this class might also be mentioned another—one, I fear, never doomed to become extinct in any age, and on whom the most labored and intelligible description of improved instruments and apparatus, from whatever source, would be lost or have but little effect, but yet, neither in numbers nor otherwise so entirely insignificant as to be passed by unnoticed. These self-styled conservatives do not as a rule take kindly to novelties, but, quite content to follow the path of writers and thinkers of the last generation, some one of whom they invariably set up to worship and accept as a guide for all time to come, could hardly be expected to become favorably impressed with any such innovation as that herein described.

Indeed so inflexible are they in adhering to obsolete habits, and so utterly incapable of freeing themselves from the grasp of preconceived notions, that anything seeming to clash with either will not be entertained for a moment.

They neither hesitate, nor, strange as it may appear, are they ashamed to declare that every structural change to which the human uterus is prone can be diagnosticated by them with the greatest facility and satisfaction by peering through a glass tube, and for all such ails their magic wand of lunar caustic is a never-failing remedy. Now, so far as this class is concerned, but little can be hoped for from anything that I might here advance; for of what benefit would be the best microscope to one who would insist on his being able to study pathalogical anatomy by the aid of a Stanhope lens?

Thus, then, on the one hand, through the well-known obstinacy of manufacturers and their workmen in persisting to carry out their own notions in spite of repeated protests, and on the other from neglect, incapacity, or other causes, on the part of practitioners, the instrument has yet to be better and more generally known before its great value can be appreciated.

There is no speculum with which I am acquainted that can be used in all cases without more or less discomfort to the patient, and the one under consideration is no exception in that respect. However, though the least objectionable of all others on this ground, and the most indispensable instrument to every gynæcologist (Dr. Sims'), may be employed to draw back the perinæum with but little pain, in the majority of cases, it is unreasonable to expect that this proceeding could be carried to an equal extent by one which, though designed for a similar purpose, can accomplish the same only by making counterpressure on the arch of the pubes and base of the bladder. But it is neither proper nor at all necessary, except in operations of more than ordinary importance, and when patients are under the influence of an anæsthetic, to insist upon such a display of the parts as this instrument is capable of affording; and on this point I have been quite explicit in the following directions for its use:

"The patient having assumed the desired position—say, on her back, with knees drawn up—and the introductory digital examination having been made, the speculum, with elevating rod drawn out, is taken in the right hand, the thumb resting on the anterior concave surface of the perineal blade, while the left index finger and thumb are used to separate the labia. It is now to be inserted downward and backward in the direction of the post-uterine cul-de-sac, and, while being thus held, the projecting handle of the elevator is to be depressed and pushed forward to the extent required to bring the uterus into a proper position in relation to the outlet, when the touch of a finger to the button-screw serves to keep everything in place.

"It will now be observed that the elevator and depressor blades describe a triangle, and the vaginal canal represents a hollow-cone, whose apex is the outlet •

"The perineal blade is now to be depressed in proportion to the amount of working space required, and, of course, with due consideration for the degree of elasticity or resistance in such case, when a turn of the set-screw will serve to secure it at any desired point.

"When the object is merely to make simple applications to the cervix, a very slight depression of the blade only is needed—rarely more than half an inch. Besides, forcible and continued traction cannot be easily tolerated, and ought to be reserved exclusively for the more important operations. In the case of patients under the influence of an anæsthetic, or where the parts have been subjected to parturient expansion, no particular exactness in this respect is called for. In others, however, the utmost care should be observed, lest profitless curiosity be appeased at the expense of a patient's comfort.

"Having thus obtained a full display of the uterus and adjacent parts, the projecting lever-rod may be removed, and the patient placed in any other than the back position, previous to or at any subsequent stage of an operation, should such a procedure be indicated.

"Indeed, I have frequently found it desirable to change the position of patients during tedious operations without removing this speculum, and in no instance have I noticed any deviation in its relation to the intra-vaginal parts from that obtained when first adjusted.

"In proceeding to remove the instrument, the steps adopted for its introduction should be reversed, the perineal blade being first released and the elevator drawn outward so that, in closing, it may clear the cervix.

"The latter purpose—closing the blades—will be best accomplished by first making slight pressure on the projecting level-rod, as in the act of elevating the anterior wall, when the button will admit of being rolled down with a touch of the finger, and the speculum can then be withdrawn."

I trust, in thus attempting to explain the manner of using and the advantages possessed by my own speculum, I shall not be understood as ignoring the merits of other such instruments, especially those of Drs. Thomas and Nott, with which I have had considerable experience, and satisfactory too; and as for that of Dr. Sims, it is hardly supposable that any gynæcologist of the present day could pretend to do without it.

The distinctive features of the instrument above described, in addition to its being self-retaining consists in its wider range of usefulness, and, unlike all other contrivances of the kind, in being capable of affording a complete display of the uterus, with ample room for all instrumental manipulation. There are, therefore, but few, if any, intra-vaginal operations in the whole range of uterine surgery, vesico-vaginal fistulæ perhaps alone excepted, but what may be conducted with the greater facility and completeness by its aid, and *without a speculum-assistant.*

However foreign to the subject of this paper the foregoing remarks may be deemed by some, I have very little doubt but that there are many who will hereafter, at least, candidly admit both their relevancy and importance.

Fig. 7 is a reversible vulsellum devised for the purpose of drawing down the uterus and maintaining it in any desired position during operations; as, for example, amputation of the cervix and extirpation of cancroid growths. To accomplish this object it is to be introduced while closed within the cervical canal, and the tenaculum points reversed by a further approximation of its fenetrated ends when it may be fastened at any degree of expansion by the ratchet attachment (See Fig. 10). I have had many opportunities of demonstrating the utility of this little instrument, and as it will also serve for a good ordinary vulsellum, I consider it an invaluable aid in most utero-vaginal opertions. It is but proper to state, however, that the principle of its mechanism is no invention of

FIG. 7.

mine, but originally suggested by examining a hinged tenaculum designed and used many years ago by my friend Dr. J. Marion Sims, though for entirely different purposes. The only original features about it, therefore, besides its adaptation to other uses, are in its having double instead of single projecting claws and reverse action.

FIG. 8.

Figs. 8 and 9 represent rake-toothed forceps employed for grasping such structures as are apt to break down readily, or yield to traction by any ordinary tenaculum or vulsellum. I have also found them especially serviceable in tearing away large masses of diffuse vegetating and other soft cancerous growths preparatory to cauterization of the subjacent tissues.

Having thus, as briefly as possible, described such an apparatus and the more important of the instruments which I have found needed in operations by galvano-cautery, I shall now submit a few cases from my clinical records selected solely on account of the intrinsic interest of each, and the instruction that may accrue from their perusal. The manner in which these cases are presented, and the accompanying illustrations, will, it is believed, render unnecessary any extended introductory remarks, or specific directions as to how such operations ought to be conducted." *Medical Record.*

## Figures and Tables

**Fig. 1. f1:**
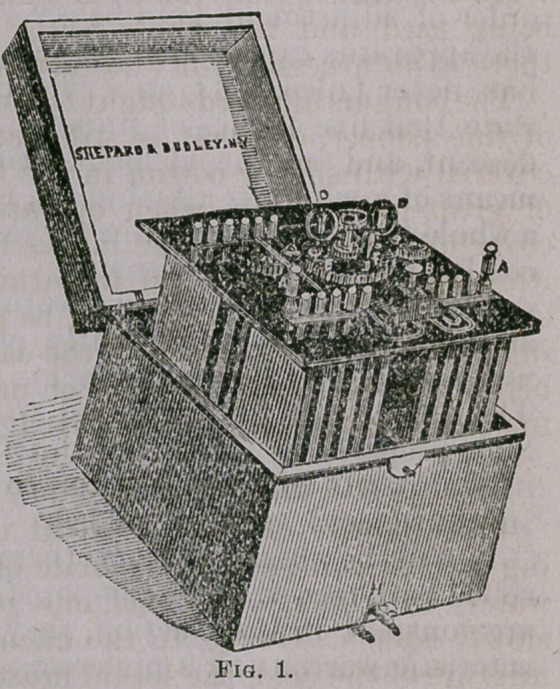


**Fig. 2. f2:**
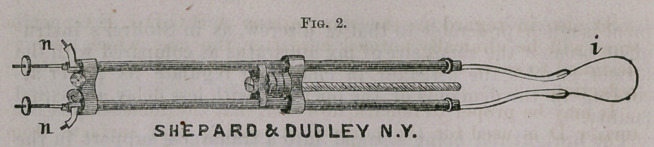


**Fig. 2. f3:**
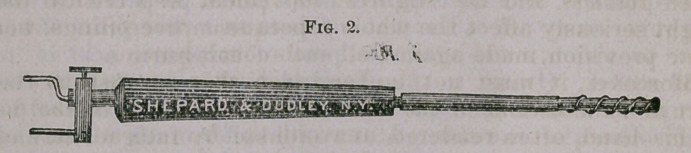


**Fig. 4. f4:**
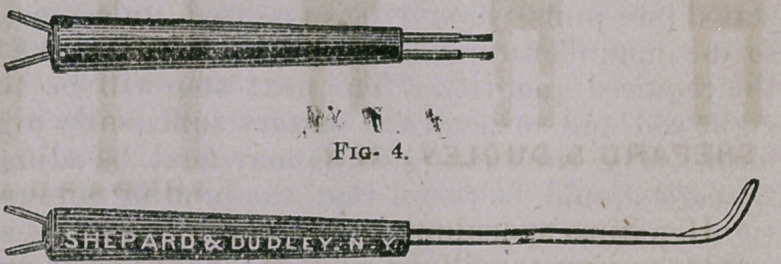


**Figure f5:**
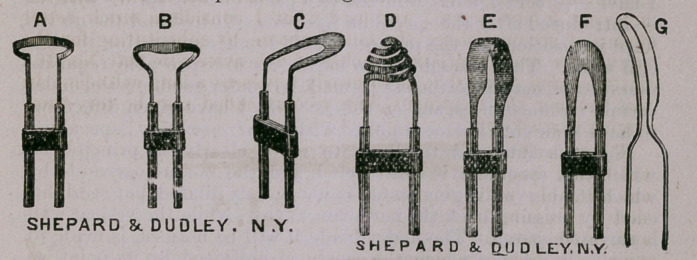


**Fig. 5. f6:**
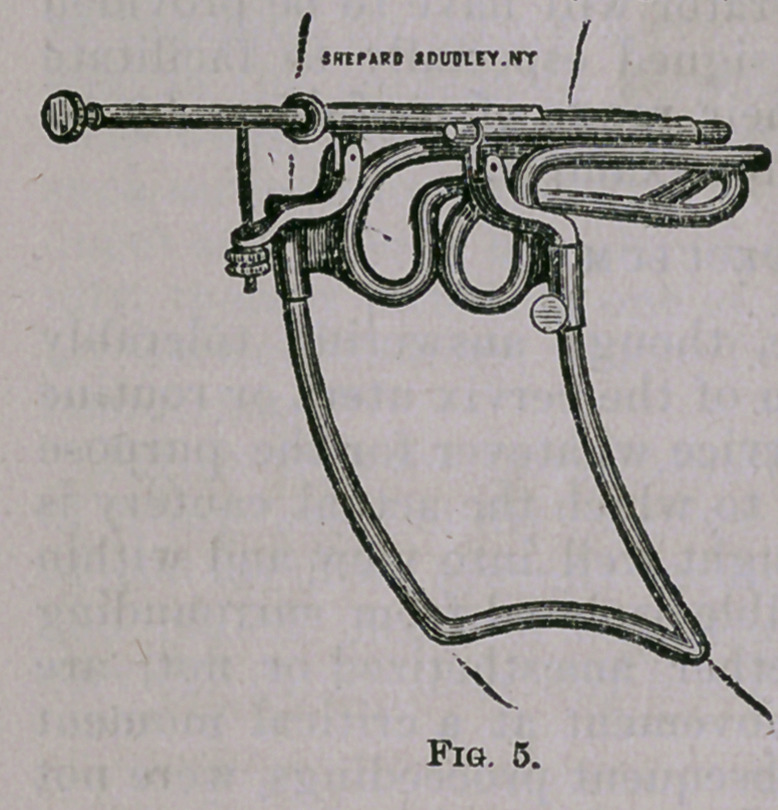


**Fig. 6. f7:**
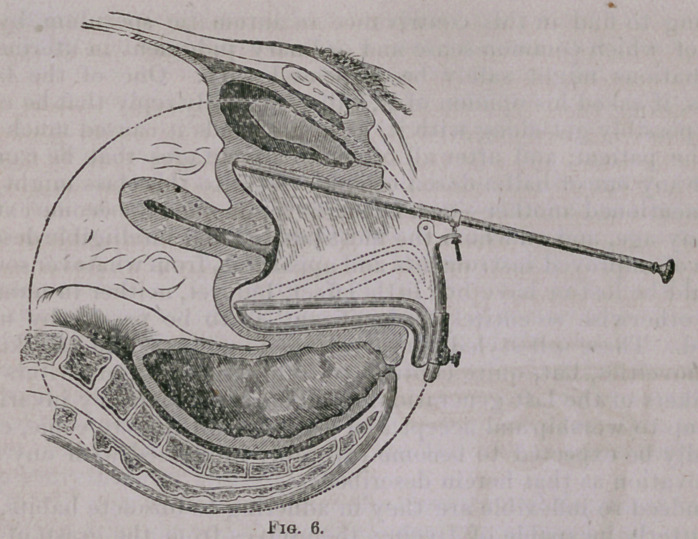


**Fig. 7. f8:**
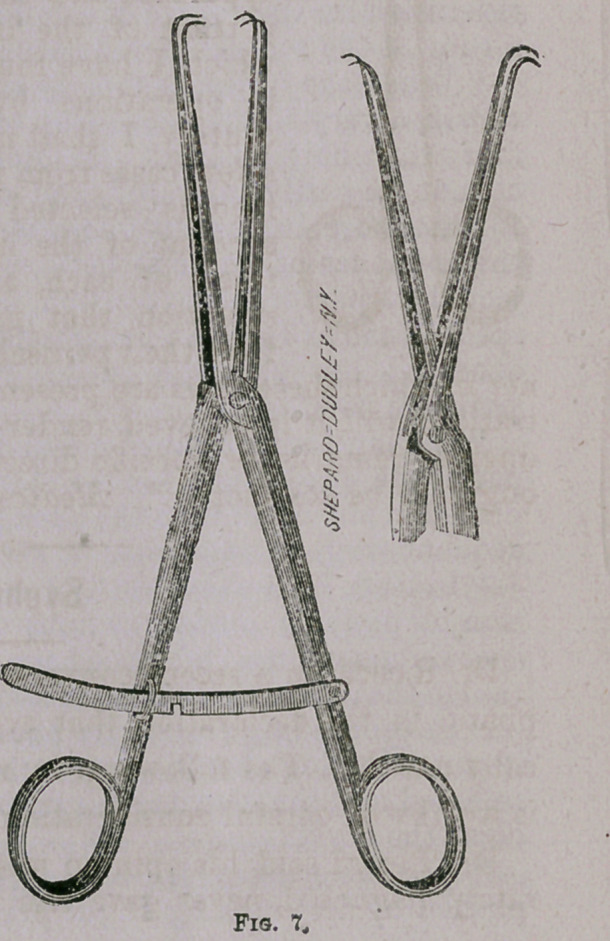


**Fig. 8. f9:**
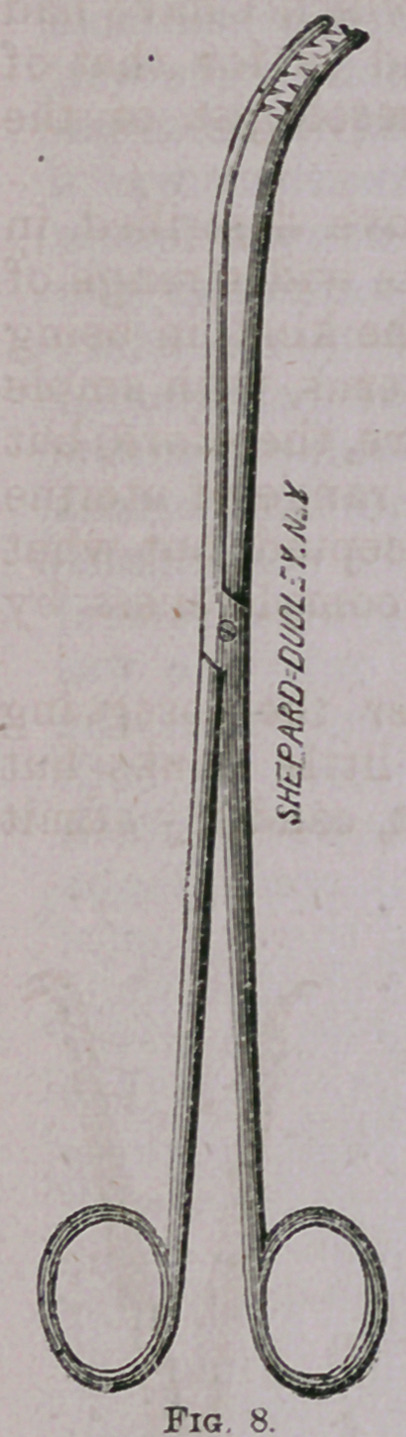


**Fig. 9. f10:**